# Project NextGen: Developing the Next Generation of COVID-19 Vaccines and Therapeutics to Respond to the Present and Prepare for the Future

**DOI:** 10.1093/cid/ciae073

**Published:** 2024-02-13

**Authors:** Kimberly A Hofmeyer, Christy L Ventura, Kimberly L Armstrong, Christopher R Houchens, Sandeep Patel, Gary L Disbrow, Robert A Johnson, Project NextGen Line of Effort Leads, Aaron C Bandremer, John H Beigel, Timothy T Belski, Ruben O Donis, Lakshmi Jayashankar, Richard A Koup, Gerald R Kovacs, Malen A Link, Karen A Martins, Robin M Mason, Sabrina M Stronsky, Daniel N Wolfe

**Affiliations:** Biomedical Advanced Research and Development Authority, Administration for Strategic Preparedness and Response, U.S. Department of Health and Human Services, Washington, DC, USA; Biomedical Advanced Research and Development Authority, Administration for Strategic Preparedness and Response, U.S. Department of Health and Human Services, Washington, DC, USA; Biomedical Advanced Research and Development Authority, Administration for Strategic Preparedness and Response, U.S. Department of Health and Human Services, Washington, DC, USA; Biomedical Advanced Research and Development Authority, Administration for Strategic Preparedness and Response, U.S. Department of Health and Human Services, Washington, DC, USA; Biomedical Advanced Research and Development Authority, Administration for Strategic Preparedness and Response, U.S. Department of Health and Human Services, Washington, DC, USA; Biomedical Advanced Research and Development Authority, Administration for Strategic Preparedness and Response, U.S. Department of Health and Human Services, Washington, DC, USA; Biomedical Advanced Research and Development Authority, Administration for Strategic Preparedness and Response, U.S. Department of Health and Human Services, Washington, DC, USA

**Keywords:** COVID-19, pandemic preparedness, COVID-19 vaccines, COVID-19 therapeutics, medical countermeasures

## Abstract

Coronavirus disease 2019 (COVID-19) epidemiology and product landscapes have changed considerably since onset of the pandemic. Safe and effective vaccines and therapeutics are available, but the continual emergence of severe acute respiratory syndrome coronavirus 2 variants introduce limitations in our ability to prevent and treat disease. Project NextGen is a collaboration between the Biomedical Advanced Research and Development Authority, part of the Administration for Strategic Preparedness and Response, and the National Institute of Allergy and Infectious Diseases, part of the National Institutes of Health, that is leveraging public–private partnerships to address gaps in the nation's COVID-19 vaccine and therapeutic capabilities. Targeted investments will advance promising next-generation candidates through the most difficult phases of clinical development to encourage further private sector interest for later stage development and commercial availability. New commercial vaccines and therapeutics that are more durable and effective across variants will improve our fight against COVID-19 and transform our response to future threats.

## Teaser

Project NextGen is a collaboration between the Biomedical Advanced Research and Development Authority and the National Institute of Allergy and Infectious Diseases that is leveraging public-private partnerships to address current gaps in the nation's coronavirus disease 2019 vaccine and therapeutic capabilities.

Operation Warp Speed (OWS) was established as a whole-of-government effort in May 2020 to execute the rapid development of medical countermeasures (MCMs) to mitigate the effects of the novel coronavirus, severe acute respiratory syndrome coronavirus 2 (SARS-CoV-2). Through multiple public–private partnerships, OWS developed, manufactured, and deployed safe and effective vaccines and therapeutics with unprecedented speed.

Years of coronavirus research drove the U.S. government vaccine candidate selection strategy that targeted the SARS-CoV-2 spike protein and use of diverse platform technologies suitable for rapid development and delivery [1, 2]. However, now, several years after the launch of OWS and the subsequent approval and distribution of first generation coronavirus disease 2019 (COVID-19) MCMs, we know more about the evolution of SARS-CoV-2, the resulting disease, and the waning immunity over time after either infection or immunization [3].

Available COVID-19 vaccines remain effective against serious illness and death; however, durability and prevention of infection and transmission against new variants is reduced [4–7]. Similarly, first-generation monoclonal antibody (mAb) effectiveness is impacted by SARS-CoV-2 viral evolution; in fact, there are currently no COVID-19 mAbs authorized for use in the United States because circulating variants escape binding [8]. This is concerning given the importance of mAbs as preexposure prophylaxis (PrEP) for people that do not respond to COVID-19 vaccines (eg, immunocompromised) as well as groups that cannot take current COVID-19 oral antivirals because of drug–drug interactions or other issues.

**Figure 1.**
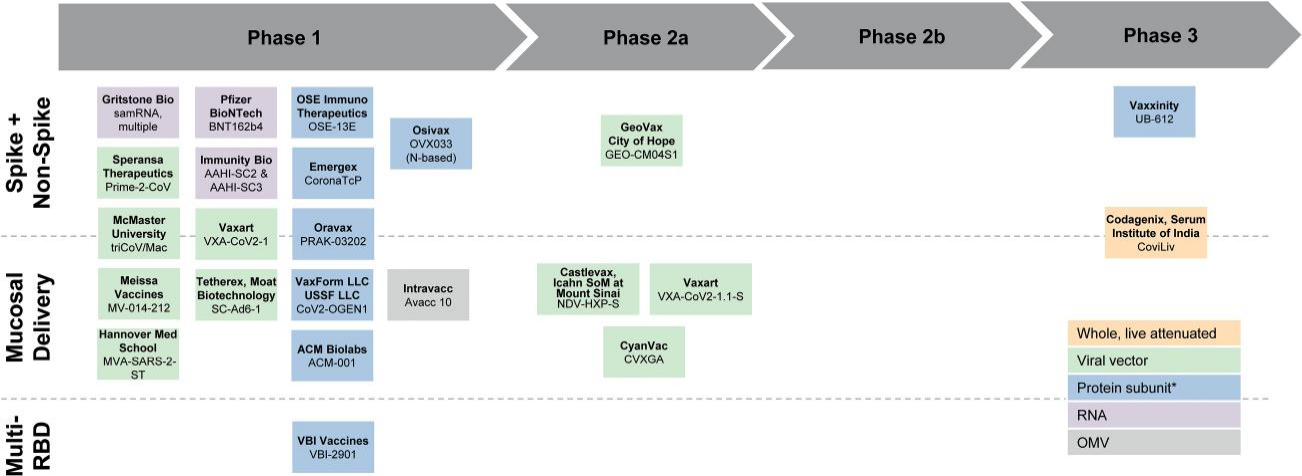
COVID-19 vaccine landscape. Representative vaccine development landscape for companies with COVID-19 candidates that align with Project NextGen criteria for candidates with mucosal administration, a spike plus non-spike antigen, or a multi-RBD antigen. Development stage noted is based on publicly available information of the most advanced trial for the candidate. *Protein subunit candidates include recombinant protein, epitope, virus-like particles (VLP; including enveloped VLPs), and nanoparticles. Abbreviations: COVID-19, coronavirus disease 2019; OMV, outer membrane vesicle; RBD, receptor-binding domain; USSF, US Specialty Formulations.

**Figure 2.**
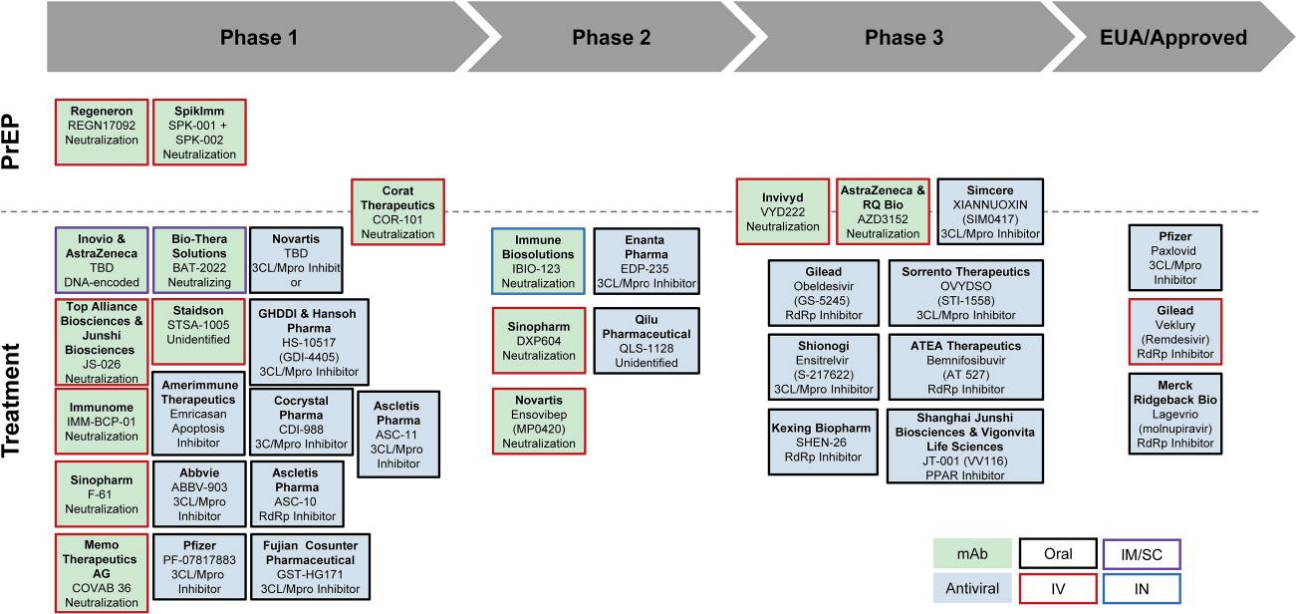
COVID-19 therapeutics landscape. Representative development landscape for companies with COVID-19 monoclonal or antiviral therapeutic candidates. The mechanism of action for each candidate is noted in the respective box. Administration route is indicated by the box border for oral; intravenous (IV); intramuscular/subcutaneous (IM/SC), and intranasal (IN). Abbreviation: COVID-19, coronavirus disease 2019.

Challenges with first-generation COVID-19 MCMs led to an increase in research focused on spike protein targets that are less prone to mutation, other nonspike viral protein targets, and alternative formulation and delivery approaches. However, for various reasons, available funding has not kept pace to advance next-generation COVID-19 MCMs through regulatory approval. Despite this slowdown in investment, there is a robust pipeline of vaccine (Figure 1) and therapeutic (Figure 2) candidates that could provide key performance improvements.

The U.S. Department of Health and Human Services and the White House COVID-19 Response Team identified these limitations and funding challenges as an opportunity to revitalize the COVID-19 vaccine and therapeutic pipeline, which, if successful, could have a significant global health impact. This became Project NextGen—a $5 billion initiative announced in April 2023 leveraging public–private partnerships to address the COVID-19 challenges of today and the future by advancing the next generation of vaccines and therapeutics. The initiative focuses on clinical development to de-risk further commercial investment for the most promising candidates. The approach strategically complements global, multisectoral work developing improved prevention and treatment solutions as well as investments in other areas, including industrial base expansion and diagnostics [9, 10].

Project NextGen includes 3 strategic areas [11]. The primary focus is SARS-CoV-2 but candidates that additionally target coronaviruses more broadly are of interest if product and program requirements are otherwise met.

- Strengthen. Advance next-generation COVID-19 vaccines that have better breadth of protection, infection and transmission blocking capabilities, and/or duration of protection against new SARS-CoV-2 variants.
- Treat. Advance next-generation COVID-19 therapeutics that have better durability against new SARS-CoV-2 variants to address current gaps in PrEP and treatments for people who do not respond to vaccination or who cannot take existing treatments.
- Enable. Advance better and innovative vaccine approaches, therapeutic platforms, and vaccine manufacturing strategies to enable faster, lower-cost production and improve efficacy and access.

## STRENGTHEN

The first generation of COVID-19 vaccines prevented an estimated 14.4 million deaths worldwide through 2021 [12]. In the United States, an estimated 18.5 million U.S. hospitalizations and 3.2 million deaths were prevented and $1.15 trillion saved through 2022, and these vaccines continue to protect against severe illness and death [5, 6, 13]. However, available vaccines have limited durability to prevent infection or transmission in the face of new SARS-CoV-2 variants, which can partially or fully escape neutralizing antibodies from prior infection or vaccination [3, 4, 14, 15].

There is a critical need to address these limitations. An improved vaccine may save more lives directly through longer duration of protection and through allowing better protection against a new strain for which the current vaccine platforms do not provide significant protection. New antigen approaches that use nonspike or multiple receptor-binding domain (RBD) antigens in vaccine formulations may improve antibody responses and induce T-cell immunity that improves durability [16, 17]. Alternative routes of administration have the potential to generate immune memory responses that are localized to the respiratory tract and may prevent infection, virus shedding, and transmission [18, 19].

A number of vaccines in the early COVID-19 development pipeline are using 1 or more of these approaches, which could potentially address some of the reasons that result in low uptake of available COVID-19 vaccines [20]. However, few vaccines have been funded to support advancement through clinical trials (Figure 1).

Project NextGen is tackling these challenges by focusing on next-generation COVID-19 vaccine candidates that are delivered via mucosal administration, use a multi-RBD strategy, or target other nonspike proteins that may strengthen vaccine breadth, durability, and transmission-blocking.

The Biomedical Advanced Research and Development Authority (BARDA) and the National Institute of Allergy and Infectious Diseases (NIAID) are implementing a coordinated strategy to achieve the goal of advancing next-generation candidates. NIAID is leveraging its clinical trial networks to advance earlier candidates into phase I/IIa safety and immunogenicity trials [21]. Existing NIAID infrastructure, network sites, and expertise enables rapid initiation of trials and increases the number of candidates that can be tested simultaneously.

In parallel, BARDA is partnering with vaccine developers to advance more mature candidates that are poised for phase IIb proof-of-concept efficacy trials [22]. Efforts will assess next-generation candidates for improved performance compared with the currently available vaccines, with a target to detect a true relative risk reduction (vaccine efficacy) of 30% or greater in the primary endpoint (virologically confirmed symptomatic COVID-19) [23]. To enable efficient study start of these phase IIb trials, 2 options are available—trials may be executed directly by developers or developers will partner with a clinical research organization under BARDA's established Clinical Studies Network, which have already started protocol development [24].

The strategy to focus on clinical development means that more candidates are supported under Project NextGen than the 1 to 2 that would be possible with an end-to-end development program through product approval and procurement. This maximizes the number of opportunities for success with what are potentially high-reward, yet-unproven, new COVID-19 vaccine approaches.

NIAID will evaluate approximately 10 next-generation candidates in phase I/IIa trials and, in parallel, BARDA will support an estimated 5 to 6 candidates through phase IIb trials. The nature of the planned public–private partnerships means that developers will in large part drive the timelines for initiating clinical trials as they manufacture product for testing and set up and execute clinical trials. Although advancement through U.S. Food and Drug Administration (FDA) licensure is outside this program, continued coordination and collaboration between BARDA/Administration for Strategic Preparedness and Response (ASPR) and the FDA, including responsiveness to emerging regulatory COVID-19 guidances, is important for program success [25, 26].

Project NextGen is also partnering to develop and validate centralized immunogenicity assays for testing of vaccine trial samples. This enables further candidate selection and meta-analyses to identify trends in vaccine responses that may not be detectable in individual studies. A deeper understanding of immune correlates of protection (COP) supports the current effort and future vaccine development for other infectious disease threats.

BARDA reviewed the COVID-19 vaccine landscape throughout the pandemic and continues to do so through Project NextGen-specific market research; work is under way to identify promising candidates.

## TREAT

COVID-19 mAbs and small molecule antivirals are critical and effective components of the pandemic arsenal that saved countless lives [27–29]. However, virus evolution has rendered previously available mAbs ineffective and there are currently no authorized mAb treatments or any PrEP options for COVID-19 [8, 30]. This gap is particularly concerning for immunocompromised people who do not respond to vaccination and may not be able to take oral antivirals because of drug–drug interactions [31–34]. The late-stage pipeline of mAbs for PrEP or treatment to address this gap is narrow in comparison to the more robust oral antiviral pipeline (Figure 2).

Project NextGen addresses a critical gap in protection options for immunocompromised people by advancing therapeutic candidates for COVID-19 PrEP. The strongest candidates are mAbs because they have a well-established path to licensure and mechanisms to achieve a long half-life, which is critical for reducing the burden of PrEP treatments on patients. BARDA is leveraging public–private partnerships to develop candidates that target spike protein epitopes with a higher barrier to resistance, such as mAbs that target epitopes that are >95% conserved among known variants. These characteristics should advance the next generation of PrEP mAbs that are more resilient against current and future SARS-CoV-2 variants and thereby more durable and commercially sustainable.

Currently available COVID-19 oral antivirals are contraindicated in some populations because of drug–drug interactions and organ toxicity [33, 34]. Fortunately, the landscape is fairly robust with COVID-19 oral antiviral candidates that have fewer drug–drug interactions in phase III clinical trials. This pipeline is driven by a combination of internal investment from the developers and National Institutes of Health (NIH)–supported phase III trials.

To complement, not duplicate, the pipeline of treatment options that could be used by those contraindicated for current oral antivirals, BARDA will partner with developers that have the potential to target earlier regulatory authorization of a new mAb therapeutic option. This could be accomplished by using unique regulatory strategies—such as leveraging previously completed phase III efficacy studies—to accelerate clinical development of the candidate while continuing to ensure safety and effectiveness.

Protecting all members of our communities and addressing the needs of vulnerable and at-risk populations is a strategic priority for all BARDA programs [35]. This principle underscores Project NextGen's strategic focus to deliver mAbs for PrEP and treatment in which they can have the largest impact, preventing and treating infections in immunocompromised people. BARDA will support advanced clinical evaluation for COVID-19 mAb candidates, whereas the development partner is responsible for manufacturing and commercialization.

Project NextGen's focus complements efforts already funded by NIH and developers to advance COVID-19 oral antivirals and the ability to predict, treat, and prevent postacute sequelae of SARS-CoV-2, including Long COVID [36]. BARDA will continue to monitor the COVID-19 therapeutic landscape and, if funding allows, could evaluate opportunities to support other gaps in the therapeutic landscape, such as next-generation oral antivirals with unique viral protein targets that offer a significant improvement over those currently in phase III trials.

## ENABLE

Rapid response to an emerging infectious disease is enabled by prior virus family research, as well as development of flexible platform technologies that can be pivoted to address the new pathogen. BARDA, NIAID, and the Department of Defense, in collaboration with industry and other partners, used this strategy successfully to develop COVID-19 MCMs under OWS [37–40].

Project NextGen is leveraging lessons learned to help advance novel long-term capabilities that can bolster preparedness and response against multiple health security threats. This includes advancing new technologies and capabilities to improve accessibility and efficacy of COVID-19 vaccines and therapeutics to enable faster, more flexible, and lower-cost production. Several of these efforts represent technologies that will break new ground from traditional approaches, requiring close interaction between developers and regulatory agencies to determine how best to move forward.

Efforts will leverage both traditional government funding mechanisms and unique authorities, like Other Transaction authorities and BARDA Ventures’ partnership with the Global Health Investment Corporation, that reach nontraditional private partners and encourage commercial investments that benefit national health security [41, 42]. This widens the Project NextGen aperture, priming the development pipeline with the best science and innovation through use of a combination of push and pull (eg, prize competitions) funding incentives. This diversified portfolio approach maximizes the chance of success for both near and long-term preparedness objectives for COVID-19 and other national health security threats.

### Next-generation Therapeutic Technologies

The COVID-19 pandemic highlighted several challenges with current approaches to rapid development of durable therapeutics. The first emergency use authorization for an oral antiviral to treat outpatient COVID-19 was not granted until almost 2 years into the pandemic despite major industry investments [43]. In contrast, the first COVID-19 mAb was authorized in the United States just 10 months after the virus sequence was released [44]. However, despite this speed, mAb platforms could not pivot quickly enough to keep pace with SARS-CoV-2 variants.

Project NextGen is supporting technologies to address these timeline and virus evolution challenges. Nucleic acid–based platforms have potential for rapid development to target diverse viral threats based on pathogen sequence data alone and may avoid the timeline and scale challenges of traditional cell-based mAb production [45]. Single-domain and multivalent antibody technologies can access more antigenic epitopes than traditional mAbs and could offer other production-related improvements [46].

Project NextGen is also supporting development of human microphysiological systems as a future capability that could potentially enable faster drug screening that is more predictive of human responses.

### Next-generation Vaccine Technologies and Capabilities

Innovations to enable faster manufacture and delivery of vaccines will advance preparedness for future outbreaks. Project NextGen is advancing on-demand manufacturing technologies that use cell-free approaches for more flexible and agile production of vaccines. A capability that is present at the point of service may also circumvent delivery and distribution delays experienced when a production facility is far from where vaccine is needed. However, to meet this ultimate goal, a variety of quality and regulatory challenges will need to be addressed. This effort begins addressing these and other technological hurdles.

Project NextGen is also enabling more robust immune COP analyses by studying capabilities that could improve the collection of samples and data from larger and more diverse populations. Better COP data could improve our understanding of the timing and need for vaccine strain changes, which could potentially enable data-driven vaccine policy recommendations specific to the needs of different populations and regulatory decisions. BARDA is supporting Decentralized Clinical Trial capabilities in which clinical visits and sampling occur at more accessible locations (eg, a retail pharmacy, at home), to improve accessibility and thereby diversity [47]. BARDA is also interested in a physiological monitoring algorithm that could alert trial organizers or participants of potential signs of illness to prompt clinical check in and infection testing. These envisioned capabilities enable the critical link between sample collection for immune analysis and correlation with disease.

### Innovative Current Good Manufacturing Practice Vaccine Manufacturing

Faster current good manufacturing practice production of vaccines at larger scales and with higher yields per manufacturing run likely means more doses are available for vaccination and at lower commercial prices. However, implementing innovative manufacturing approaches is often prohibitive. For late-stage candidates or licensed vaccines, the incorporation of new approaches into an existing production line carries cost and regulatory risks. For earlier stage candidates, there may be challenges in finding a manufacturing partner with the appropriate capabilities or the funds to procure the required dedicated equipment or parts.

Project NextGen will provide support and incentives to overcome current good manufacturing practice hurdles, with the aim of enabling vaccine developers to improve the yield, scale, speed, or cost. Innovative technologies could encompass all aspects of manufacturing, from excipients and other materials that may facilitate better production, to upstream and downstream processing, through final formulation. There is particular interest in new approaches that move away from traditional needle/syringe administration, novel recombinant antigen production and purification approaches, and formulations that significantly increase the vaccine immune response relative to the existing formulation.

## NEXT STEPS

Project NextGen is well under way and supported by the following integrated activities that will run in parallel throughout the effort.

### Conducting Stakeholder Engagement

Project NextGen is expected to have a broad impact on the COVID-19 MCM landscape. Thoughtful program and product development that enables long-term success requires engaging with end users who will administer MCMs and the communities that will receive those products.

BARDA and NIAID have augmented prior market research with direct input from product developers through a variety of Requests for Information and 1-on-1 engagements via BARDA TechWatch [48]. BARDA has also engaged with external organizations to understand product attributes that enable increased uptake and access. A recent example is the efforts of the National Biodefense Science Board, a Federal Advisory Board to ASPR, that provided important recommendations for prioritizing product attributes to maximize access for next-generation COVID-19 vaccine and therapeutics [49].

Stakeholder discussions continue to gain further perspectives on appropriate consideration that could be provided by awardees to the U.S. government and taxpayers if Project NextGen investments result in regulatory approval and commercialization. Considerations require balancing return on investment, including the life-saving and economic benefits of MCMs, with ensuring proper incentives to take products to licensure to meet public health needs.

### Releasing Funding Announcements and Opportunities

Transparency, including ensuring visibility of clearly articulated funding opportunities, is an ongoing priority that is critical to Project NextGen success. BARDA routinely updates its Broad Agency Announcement and other funding opportunities, including Project NextGen priorities, and publicizes updates through multiple avenues [50]. NIAID's Project NextGen website describes its vaccine candidate selection process for phase I/IIa trials as well as non-Project NextGen opportunities for COVID-19 vaccines [51].

### Supporting Product Development

Project NextGen includes the supportive benefits of partnering with BARDA or NIAID. The program will provide both nondilutive capital to industry partners, providing funding to advance products without the requirement to give up ownership, and also product development expertise, support capabilities, and access to interagency relationships. Technical expertise and support are fundamental to BARDA's success in public–private partnerships, which have currently delivered 86 approvals, licensures, or clearances of MCMs through the FDA [52].

Project NextGen announced more than $1.4 billion of funding across all 3 lines of effort in August 2023, and more than $500 million for vaccine candidates, trial support, and enabling technologies in October 2023 [24, 53].

## CONCLUSION

Project NextGen is focused primarily on generating clinical data to advance improved COVID-19 vaccines and therapeutics and enhance the nation's and global preparedness for future pandemic threats. It is a historic opportunity to break through barriers and address challenges to develop the next generation of COVID-19 vaccines and therapeutics.

The bedrock of this program is the U.S. government's goal to revitalize the pipeline of solutions to address the most pressing gaps in COVID-19 prevention and treatment capabilities for the nation overall and, critically, for the people most vulnerable to the effects of this infectious disease.

## Notes

***Acknowledgments***. The authors appreciate the input of Frank Arnold, Carl Burke, Huyen Cao, Christopher Dale, Joe Gerstner, Edward McGurrin, Mark Michalik, Courtney Petro, Kathryn Reid, Rebecca Reindel, Shyam Rele, and George Robertson.

***Financial support*.** The author(s) reported there is no funding associated with the work featured in this article.

***Project NextGen Line of Effort Leads*.** Aaron C. Bandremer (Biomedical Advanced Research and Development Authority, Administration for Strategic Preparedness and Response, U.S. Department of Health and Human Services); John H. Beigel (Division of Microbiology and Infectious Diseases, National Institute of Allergy and Infectious Diseases, National Institutes of Health); Timothy T. Belski (Biomedical Advanced Research and Development Authority, Administration for Strategic Preparedness and Response, U.S. Department of Health and Human Services); Ruben O. Donis (Biomedical Advanced Research and Development Authority, Administration for Strategic Preparedness and Response, U.S. Department of Health and Human Services); Lakshmi Jayashankar (Biomedical Advanced Research and Development Authority, Administration for Strategic Preparedness and Response, U.S. Department of Health and Human Services); Richard A. Koup (Vaccine Research Center, National Institutes of Health); Gerald R. Kovacs (Tunnell Government Services, Inc.); Malen A. Link (Biomedical Advanced Research and Development Authority, Administration for Strategic Preparedness and Response, U.S. Department of Health and Human Services); Karen A. Martins (Biomedical Advanced Research and Development Authority, Administration for Strategic Preparedness and Response, U.S. Department of Health and Human Services); Robin M. Mason (Biomedical Advanced Research and Development Authority, Administration for Strategic Preparedness and Response, U.S. Department of Health and Human Services); Sabrina M. Stronsky (Biomedical Advanced Research and Development Authority, Administration for Strategic Preparedness and Response, U.S. Department of Health and Human Services); Daniel N. Wolfe (Biomedical Advanced Research and Development Authority, Administration for Strategic Preparedness and Response, U.S. Department of Health and Human Services).
